# Endothelial activation and damage as a common pathological substrate in different pathologies and cell therapy complications

**DOI:** 10.3389/fmed.2023.1285898

**Published:** 2023-11-14

**Authors:** Marta Palomo, Ana Belén Moreno-Castaño, María Queralt Salas, Silvia Escribano-Serrat, Montserrat Rovira, Elena Guillen-Olmos, Sara Fernandez, Helena Ventosa-Capell, Lina Youssef, Fatima Crispi, Meritxell Nomdedeu, Julia Martinez-Sanchez, Blanca De Moner, Maribel Diaz-Ricart

**Affiliations:** ^1^Hemostasis and Erythropathology Laboratory, Centre de Diagnòstic Biomèdic, Hospital Clínic de Barcelona, Institut de Recerca August Pi Sunyer, University of Barcelona, Barcelona, Spain; ^2^Hematology External Quality Assessment Laboratory, Centre de Diagnòstic Biomèdic, Hospital Clínic de Barcelona, Barcelona, Spain; ^3^Hematopoietic Stem Cell Transplantation Unit, Hematology Department, Institute of Cancer and Blood Diseases, Hospital Clínic de Barcelona, Institut de Recerca August Pi Sunyer, Barcelona, Spain; ^4^Department of Nephrology and Kidney Transplantation, Hospital Clínic de Barcelona, Centro de Referencia en Enfermedad Glomerular Compleja del Sistema Nacional de Salud (CSUR), University of Barcelona, Barcelona, Spain; ^5^Medical Intensive Care Unit, Hospital Clínic de Barcelona, Barcelona, Spain; ^6^BCNatal – Barcelona Center for Maternal Fetal and Neonatal Medicine, Hospital Clínic de Barcelona and Hospital Sant Joan de Déu, Institut de Recerca August Pi Sunyer, University of Barcelona, Barcelona, Spain; ^7^Josep Carreras Leukaemia Research Institute, Hospital Clinic, University of Barcelona, Barcelona, Spain; ^8^Centre for Biomedical Research on Rare Diseases (CIBER-ER), Madrid, Spain; ^9^Hemostasis and Hemotherapy Department, Institute of Cancer and Blood Diseases, Hospital Clínic de Barcelona, Barcelona, Spain

**Keywords:** endothelial damage, cardiovascular disease, chronic kidney disease, obesity, major depression, sepsis, COVID-19, hematopoietic stem cell transplantation

## Abstract

The endothelium is a biologically active interface with multiple functions, some of them common throughout the vascular tree, and others that depend on its anatomical location. Endothelial cells are continually exposed to cellular and humoral factors, and to all those elements (biological, chemical, or hemodynamic) that circulate in blood at a certain time. It can adapt to different stimuli but this capability may be lost if the stimuli are strong enough and/or persistent in time. If the endothelium loses its adaptability it may become dysfunctional, becoming a potential real danger to the host. Endothelial dysfunction is present in multiple clinical conditions, such as chronic kidney disease, obesity, major depression, pregnancy-related complications, septic syndromes, COVID-19, and thrombotic microangiopathies, among other pathologies, but also in association with cell therapies, such as hematopoietic stem cell transplantation and treatment with chimeric antigen receptor T cells. In these diverse conditions, evidence suggests that the presence and severity of endothelial dysfunction correlate with the severity of the associated disease. More importantly, endothelial dysfunction has a strong diagnostic and prognostic value for the development of critical complications that, although may differ according to the underlying disease, have a vascular background in common. Our multidisciplinary team of women has devoted many years to exploring the role of the endothelium in association with the mentioned diseases and conditions. Our research group has characterized some of the mechanisms and also proposed biomarkers of endothelial damage. A better knowledge would provide therapeutic strategies either to prevent or to treat endothelial dysfunction.

## 1. Introduction

At the conference on “Women and Science” organized by the European Parliament and Commission in April 1998 in Brussels, both institutions agreed in a formal statement on the “need to identify efforts to increase the presence of women in research in Europe.” Actually, as the scientific career advances, the proportion of women decreases (1). In our case, we are proud to constitute a group of multidisciplinary scientific women who address the same exciting topic: endothelial activation and damage in association with different pathologies and as a consequence of certain therapies.

For years, the vascular endothelium was considered an inert barrier, but, nowadays, it plays a fundamental role in human health and disease. This biologically active interface is constituted by 1 to 6 × 10^13^ endothelial cells (ECs) in an adult human being and covers a large surface area (between 4,000 and 7,000 m^2^) (2–4). The endothelium has a huge range of functions (5), including the maintenance of the vascular homeostatic balance, modulation of the vascular tone, participation in the recruitment of immune cells, and the generation of new blood vessels, among others.

All these functions are differentially regulated in space and time, showing the heterogeneity of the ECs in different organs in terms of morphology, structure, and barrier function (6, 7). The pulmonary endothelium is localized at a crucial interface and it is formed by an heterogenous cell monolayer that acts as a selective barrier between blood, airways, and lung parenchyma (8). Blood vessel endothelium crosses every tissue, exhibiting unique structural and functional properties in each vascular bed. As a result of organ-specific requirements, the vascular system varies in its organization and specifically in cell-to-cell junctions, which are crucial in the integrity of blood vessels, depending on the anatomical site. While tight junctions are well organized in arteries and brain microvessels, they are more unstructured in veins, capillaries, and organs where a higher rate of exchange is needed (9). Due to their location, the endothelium is directly exposed to all physiological and pathological stimuli (10, 11). These cells are able to adapt to a wide range of environmental conditions, however, noxious stimulus induce local or systemic endothelial activation (12).

Endothelial cell activation and damage imply a range of phenotypic changes in the endothelium and differ according to many physiological variables. If the activation stimuli are persistent in time and/or intense enough, the endothelium may become dysfunctional causing abnormal functional and structural changes, being the main consequence of the loss of vascular integrity with the detachment of ECs exposing a more thrombogenic extracellular matrix (ECM) (13). The relevance of endotheliopathy in the progression of several diseases has been increasingly accepted in the scientific community. The present review aims to summarize the complexity of this process in a range of pathological situations that share endothelial damage (ED) as a common feature.

**FIGURE 1:**
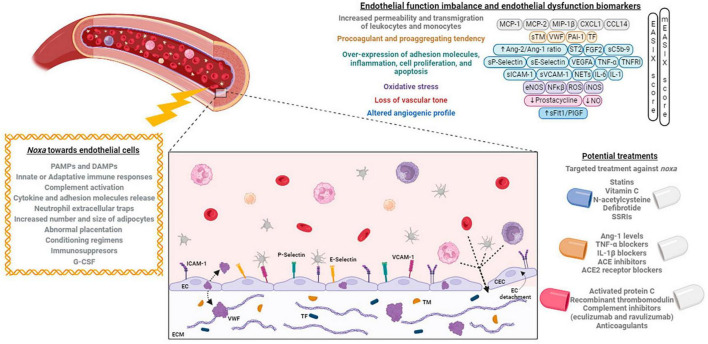
Noxa, mechanisms, and biomarkers involved in endothelial dysfunction, and its potential treatment. Different factors alter the endothelial cells phenotype causing an imbalance that can be identified by the expression of different biomarkers. At right, the principal developed treatments targeting the endothelium are exposed. Created with BioRender (available from https://www.biorender.com).

**FIGURE 2:**
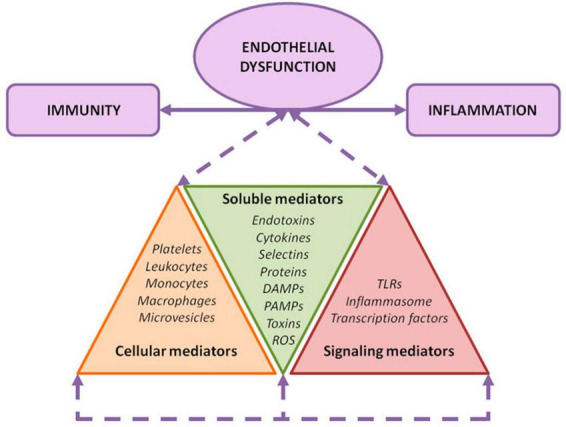
Endothelial damage mechanisms. Exposure of the endothelium to different noxa leads to a convergence among inflammation, immunity, and endothelial activation. As depicted, several soluble, cellular, and signaling mediators are involved in this orchestrated response.

Historically, our team initiated the research on endothelial activation and damage in the context of end-stage chronic kidney disease (CKD). Further collaboration with other teams in the Hospital Clinic and Institut de Recerca August Pi Sunyer helped us to expand the investigation into other pathologies with associated cardiovascular risk, such as obesity and major depression. A joint venture with obstetricians followed to explore the role of the endothelium in pregnancy-related pathologies, such as preeclampsia (PE), in which the dysregulation of the complement system plays a crucial role. Moreover, a tight partnership with the intensive care unit (ICU) prompted us to explore the ED in septic syndromes and how the severity of the disease could have a gradual impact on the endothelium. Then, with the global outbreak of the coronavirus SARS-CoV-2 that caused the COVID-19 pandemic starting in 2020, our efforts were focused on characterizing the associated endotheliopathy. Furthermore, in collaboration with the hematopoietic stem cell transplantation (HCT) unit at the Hospital Clínic, we were one of the first groups to demonstrate the role of the ED as a pathological substrate for the complications appearing in early post-transplantation. We are now progressing in the research of the role of the endothelium in the development of severe complications that may compromise the promising curative potential of new therapies, such as the use of chimeric antigen receptor T (CAR-T) cells. The investigation performed during these years to explore the mechanisms involved has already provided us with diagnostic and prognostic biomarkers and potential therapeutic targets. Future research should still generate additional tools focused on protecting the endothelium (Figures 1, 2).

## 2. Endothelial activation and damage in end-stage chronic kidney disease

Chronic kidney disease is a major public health issue with an increasing prevalence. It is associated with poorer quality of life, reduced life expectancy, and, therefore, high rates of morbidity and mortality, and increased hospitalization costs (14, 15). An increment in cardiovascular diseases has been highlighted as the main cause of the increased morbidity and mortality in this population; however, it cannot be fully explained by the presence of traditional cardiovascular risk factors. Endothelial activation and damage in CKD patients have been described as related to sustained toxic, oxidative stress, and inflammatory conditions. Endothelial dysfunction has been proposed as a pathophysiological substrate for accelerated atherothrombosis, hemostasis alterations, inflammatory activity, and impaired immune response in these patients (16, 17).

Endothelial activation in CKD may be attributed to different factors: the pulsatile blood flow and disturbed shear stress (18), the presence of uremic toxins, such as indoxyl sulfate, and the production and release of oxidative stress and inflammation related products to the circulation (19). All these elements constitute the uremic environment, and could be classified into three major categories of mediators: (i) soluble, (ii) cellular, and (iii) signal transduction mediators (19).

Innate immune system alterations have been also reported in association with end-stage renal disease, aggravated by dialysis procedures. In addition, CKD is related to gut dysbiosis, with a significant loss of the gut microbiota diversity due to the uremic condition, dietary restrictions, administered drugs (antibiotics, phosphate binders, and oral iron supplementation), and hypervolemia, leading to intestinal wall congestion and edema (20, 21). In addition to the impaired renal function, there is also a reduced function of the intestinal barrier with increased permeability to different size molecules. All together enrich the toxic milieu in uremia

There is *in vivo* and *in vitro* evidence demonstrates endothelial activation and damage in association with CKD, causing impaired endothelium-dependent vasodilatation and increased plasma levels of circulating cell adhesion molecules, such as intercellular adhesion molecule 1 (ICAM-1), vascular cell adhesion molecule 1 (VCAM-1), and E-selectin (22–24). Also, other ED-derived proteins, such as monocyte chemoattractant protein-1 (MCP-1) (25), angiopoietin-2 (Ang-2) (26), tissue factor (TF) (27–29), and total von Willebrand factor (VWF) (30, 31) are elevated in circulation. Endothelial activation is considered an early trigger for atherosclerosis and, therefore, in the setting of CKD may explain the increased cardiovascular risk in this population, beyond traditional cardiovascular risk factors (32).

Our research group has carefully studied the ED in uremia by exposing ECs to culture media supplemented with sera from patients on renal replacement therapy. ECs exhibited alterations in their morphology, with accelerated proliferation (33). They also showed signs of inflammation, expressing VCAM-1, ICAM-1 on their surface, with activation of the intracellular protein p38MAPK (34). The ECM produced by these ECs was characterized by a less intricate network of fibrils (27) and an increased thrombogenicity, mainly due to a higher expression of TF (27), VWF (34), and thrombomodulin (TM). No changes in ADAMTS-13 activity, the VWF metalloprotease, were detected in patients’ plasma (35).

The analysis of the proteome of ECs grown under uremic conditions versus control (36) provided information on the differentially expressed proteins. Also, antioxidant enzymes, such as glutathione peroxidase, superoxide dismutase, and peroxiredoxin, were detected to be increased, suggesting an adaptive response to the oxidative stress induced by uremic media. Most of the proteins found to be unregulated in the uremic ECs are related to nuclear factor kappa B (NF-κB) (35). This protein is key in mediating inflammatory and immunological responses and oxidative stress. In addition, elements participating in innate immunity, such as Toll-like receptor 4 and the inflammasome nucleotide-binding oligomerization domain-like receptor prying domain-containing-3 (NLRP3, also known as NALP3) were also upregulated in ECs exposed to the uremic milieu (37). ED can also develop into apoptotic changes (38, 39).

Therefore, endothelial activation is associated with inflammation, oxidative stress, and alterations of immune mechanisms in CKD patients. Therapies focused on protecting the endothelium at different levels could diminish the accelerated cardiovascular risk in this population.

## 3. The endothelium in obesity

Obesity is a chronic systemic metainflammation. Is associated with oxidative stress, endothelial dysfunction, and vasculopathy, and, therefore, constitutes an important risk factor for atherothrombotic cardiovascular morbidity and mortality (40, 41). In addition, obesity is related to dyslipidemia, hypertension, insulin resistance, and diabetes mellitus, which are conditions that also have a deleterious impact on the endothelium.

The increase in the number and size of adipocytes appears to be the initial event of adipose tissue dysfunction, resulting in hypoxia and defects in the lipids accumulation, together with infiltration of inflammatory macrophages, the switch of adipose tissue-resident macrophages to a proinflammatory phenotype, and conversion of preadipocytes to macrophages. Activation of non-adipocyte stromal cells and secretion of factors from the adipose tissue lead to an increased presence of chemokines and cytokines in plasma, which may participate in the development of chronic inflammation, angiogenesis, and atherothrombotic changes (42–46). There is evidence of the secretion of cytoadipokines from different adipose tissue depots (42, 47–49).

In studies performed by our group, cultured ECs were exposed to the secretome of adipose tissue from visceral and subcutaneous locations of obese and non-obese individuals (50). The cytokines secreted by the adipose tissue of obese subjects caused an adverse effect on the cultured ECs (50), characterized by increased proliferation, morphology alteration, higher expression of VCAM-1, ICAM-1, and VWF, and production of a more thrombogenic ECM. The visceral secretomes induced the strongest expression of these markers, which occurred through NF-κB activation in ECs, together with an increased presence of proinflammatory cytokines (interleukin-6, IL-6), and neutrophil and monocyte chemo-attractants (MCP-1, MCP-2, MIP-1β, CXCL1, and CCL14), in the secretomes from obese adipose tissue (42–47). All these alterations could be involved in vascular and systemic aging (51, 52). Our findings are in agreement with observations by several groups of a better cardiovascular risk profile for those obese individuals with low levels of visceral adipose tissue, known as “healthy obesity.”

We believe that excessive fat accumulation causes activation of the stromal cell fraction, altering the adipose tissue secretion pattern. The synergic proinflammatory and prothrombotic profiles of the obese secretome are responsible for the systemic macrovascular endothelial activation observed in obesity.

## 4. Major depression and cardiovascular risk: role of the endothelium

Major depression and cardiovascular disease are two comorbid conditions highly prevalent that constitute an important health concern in developed countries Signs of ED have been demonstrated in major depression (53, 54). In this regard, inflammation and ED are mechanisms potentially connecting depression to cardiovascular disease (55–57).

Our group was able to demonstrate significant elevation in circulation of different biomarkers of ED, such as circulating endothelial cell (CEC), VWF, and soluble VCAM-1, in patients with the diagnose of major depression (58). Moreover, treatment with the selective serotonin reuptake inhibitor (SSRI) escitalopram exhibited a protective role since biomarker levels decreased substantially in a gradual manner. In addition, when cultured ECs were exposed to the sera from these patients, these findings were reproducible. ECs exhibited signs of inflammation, oxidative stress, and increased thrombogenicity of the ECM generated, which were inhibited significantly by the presence of escitalopram *in vitro* (58).

There is strong evidence on the role of serotonin in the cardiovascular system. Platelets, the main carriers of serotonin (59), play a key role in the development of cardiovascular events. In experimental studies performed by our group, exogenous addition of serotonin to blood samples potentiated platelet functions, increasing their procoagulant behavior, and enhancing thrombus formation on damaged vascular surfaces, effects that were inhibited by the presence of SSRIs (60, 61). In addition, in experiments performed with blood samples from patients with major depression, a pronounced procoagulant profile, with increased platelet thrombus and fibrin formation, was observed at the moment of diagnosis and normalized after 24 weeks of treatment with escitalopram (62). Altogether, our results suggest that both platelets and endothelium are two key hemostatic components, whose responses may be altered and may be acting synergistically in major depression (63).

## 5. Endothelial damage in pregnancy-associated complications

Preeclampsia is a life-threatening pregnancy-associated disorder that affects 2–8% of pregnancies (64). It is defined as new-onset hypertension with other signs of endothelial systemic damage, accompanied by signs of end-organ damage, such as proteinuria, acute kidney failure, liver dysfunction, hemolysis or thrombocytopenia, occurring after 20 weeks of gestation (65). PE is a heterogenous disorder, with a large variability in its associated risk factors and clinical presentation (66). The exact pathophysiology remains unclear despite exhaustive investigation (67). However, the most accepted hypothesis is known as the two-stage model claiming that complications originate during abnormal placentation at the beginning of pregnancy, followed by generalized inflammation and progressive maternal ED (68). Resulting placental insufficiency and overt clinical signs of PE do not manifest usually until the last pregnancy trimester (69) with a possibly rapid and unexpected progression from mild to severe PE (70). Unfortunately, there is no effective treatment and delivery if the only available intervention (71).

Endothelial dysfunction has been accepted as one of the key mechanisms in PE development (72). In normal pregnancy, the uterine vasculature undergoes significant adaptations to ensure proper blood supply to the developing fetus (73). These adaptations mainly involve increased vasodilation and decreased vasoconstriction, allowing for higher blood flow to the uterus (73). However, these adaptations are disrupted in PE, leading to vasoconstriction and inadequate blood supply to the placenta and fetus (74).

The dysfunctional endothelium promotes the production and release of pro-inflammatory cytokines (75), such as tumor necrosis factor-alpha (TNF-α), interleukin 6 and 1b (IL-6 and IL-1b), and adhesion molecules (VCAM-1 and ICAM-1) (76). The cytokines activate immune cells, causing excessive inflammatory responses (77), and the adhesion molecules promote the breakdown of EC-cell contacts (78). Prolonged ECs activation results in a cycle of inflammation and oxidative stress. This inflammatory environment contributes to the extensive ED and organ dysfunction seen in PE, including kidney, liver, and brain involvement (79). Furthermore, this ED is associated with a dysregulation of the complement system (80). The maternal innate immune system is crucial throughout pregnancy, providing protection against pathogens while inducing tolerance to semi-allogeneic fetal and placental development (81). Dysregulation of the maternal immune system during PE leads to overstimulation of the complement system as a compensatory mechanism (82), with recruitment of phagocytic cells and neutrophils to the site of stimulation (83). This phenomenon manifests with elevated plasma C5b9 in PE mothers and C5b9 deposits on ECs (76, 83).

In addition, there is imbalance in the coagulation system (80). Damaged endothelium does not produce sufficient anticoagulant factors, such as tissue factor pathway inhibitor (TFPI), and TM (84), in association with increased VWF (85). This prothrombotic state increases the risk of thrombosis and microvascular fibrin deposition, further impairing placental blood flow (86) and contributing to the development of maternal organ dysfunction (87).

Angiogenic factors have emerged as the most specific biomarkers of PE ever described and have recently been incorporated as essential components in the prediction, diagnosis and prognosis of PE (88). A proper angiogenic balance during pregnancy is critical for adequate development of fetus and placenta together with appropriate maternal cardiovascular adaptation to pregnancy (89). Indeed, angiogenic factors are essential not only for new vessel formation but also to keep the maternal endothelium healthy by promoting vasorelaxation, adequate permeability and cell survival (90). In PE, placental inflammation and increased oxidative stress cause release of larger amounts of sFlt1 over PIGF (91), with an antiangiogenic profile reflecting placental malfunctioning and maternal endothelial dysfunction (92).

In conclusion, endothelial dysfunction is key in the pathogenesis of PE. Impaired NO production, increased vasoconstriction, inflammation, innate immunity dysregulation, and coagulation and angiogenic imbalance contribute to the hypertension, poor placental perfusion, and multiple organ damage occurring in this condition. Other pathways, like altered lipid metabolism (93), mitochondrial dysfunction (94), and maternal response to circulating trophoblast-derived extracellular vesicles (95) may be also involved. Furthermore, endothelial dysfunction seems to act as a cardiometabolic stressor that may culminate in long-term cardiovascular complications in women who developed PE during pregnancy (96). Further elucidation of the molecular mechanisms involved is critical for the development of potential therapeutic strategies aim at preventing or reducing the adverse consequences associated with this syndrome.

## 6. Endothelial alterations in septic syndromes

Sepsis is a life-threatening organ dysfunction caused by a dysregulated host response to infection (97). The endothelium orchestrates a beneficial local host response to infection by regulating the vasomotor tone, leukocyte trafficking, vascular permeability and hemostasis. However, when the response is overproduced, a systemic and untargeted dysregulated inflammatory response leads to endothelial hyperactivation, resulting in tissue hypoperfusion and subsequent multi-organ failure and death. In the lung, this translates as a localized injury to the alveolar-capillary membrane, fostering the onset of acute respiratory distress syndrome (ARDS). ARDS is characterized by an acute onset of respiratory failure typically requiring mechanical ventilation and radiographic bilateral pulmonary opacities of non-cardiogenic origin. Direct ARDS occurs after a direct insult to the lung tissue, leading to an increase in the capillary hydrostatic pressure and interstitial and alveolar flooding, impaired gas exchange, and decreased lung compliance. Indirect ARDS is triggered by a systemic insult and the release of inflammatory mediators that eventually damage the pulmonary endothelium. Its most common cause is sepsis (98).

Activation of the endothelium in sepsis occurs directly by recognition of pathogen-associated molecular patterns (PAMPs) through patron recognition receptors, such as Toll-like receptors (TLRs) expressed in ECs, and, indirectly, by released proinflammatory cytokines, such as TNF-α, IL-6, and IL-1, complement components or neutrophil extracellular traps (NETs) (99). Damage-associated molecular patterns (DAMPs) can also be recognized by ECs, contributing to the amplification of the inflammatory cascade. This endothelial activation leads to a proadhesive, proinflammatory, prothrombotic, and proapoptotic phenotype.

The endothelial barrier integrity is altered during sepsis due to the action of inflammatory mechanisms, such as metalloproteinases and heparanase, causing glycocalyx deconstruction and disruption of ECs junctions, and increasing endothelial permeability, albumin extravasation, and capillary leak (100). Glycocalyx degradation may be potentially compounded by fluid resuscitation practices (101). In addition, there is dysregulation of the NO pathway with a decreased activity of the endothelial nitric oxide synthase (eNOS) and an increased NO production by inducible nitric oxide synthase (iNOS), altering the vascular vasomotor tone toward vasodilatation and producing reactive nitrogen species (102). Moreover, infection triggers ECs to produce ROS, leading to EC apoptosis and production of proinflammatory cytokines, acting not only as a victim but also as an active participant and amplifier of the inflammation. This proinflammatory environment increases expression of adhesion molecules (VCAM-1, ICAM-1, and selectins) on the ECs surface, promoting subsequent leukocyte rolling, adhesion and transmigration, contributing to inflammation and progression of endothelial dysfunction (103). The activated endothelium also interacts with platelets, which are activated directly by PAMPs or indirectly by innate immune cells, promoting a prothrombotic phenotype in which an increased production of VWF seems to play a major role. ADAMTS-13 activity has been found to be decreased in patients with sepsis, which may increase platelet-vessel wall interaction by an increased presence of ultra-large VWF multimers (104, 105). Furthermore, activated platelets also contribute to inflammation by releasing proinflammatory proteins that help establish a “cross-talk” with the endothelium (106). Indeed, thrombocytopenia is frequently seen in sepsis, probably due to excessive peripheral consumption, and it is associated with increased disease severity and mortality (107).

Upon sepsis-induced endothelial activation, there is also an increased expression of TF with subsequent extrinsic coagulation pathway activation. This procoagulant state is favored by a dysregulation of the endothelial anticoagulant and fibrinolytic properties, with decreased protein C activation, TM and TFPI and increased PAI-1 release. This procoagulant state favors thrombosis in the microvasculature, potentially causing disseminated intravascular coagulation (DIC), which is associated with poor prognosis in patients with sepsis (108, 109).

Neutrophil extracellular traps have also an important role in sepsis-induced endothelial dysfunction, causing the expression of adhesion molecules, participating in the prothrombotic state by increasing platelet adhesion on the ECs surface, and contributing to thrombin-mediated fibrin generation (110, 111).

This ED significantly alters the microcirculation, with decreased vascular flow and subsequent organ hypoperfusion, with mitochondrial dysfunction leading to organ failure and death. Experimental and clinical studies have demonstrated a correlation between endothelial dysfunction and sepsis severity, highlighting the crucial role of the endothelium in the pathophysiology of sepsis-induced organ dysfunction, and arising as an attractive therapeutic target (112–114). Thus, in the last decades, experimental and clinical studies have strived to find effective treatments targeting sepsis-induced endothelial dysfunction (111), however, none of them have shown survival improvement in large randomized clinical trials. Our group has demonstrated the utility of an *in vitro* model of ED in sepsis, able to show a gradual effect depending on the severity of the disease, which may constitute a useful tool to explore different treatments.

## 7. The endotheliopathy developed in COVID-19

The COVID-19 pandemic, caused by the emergence and worldwide spread of the SARS-CoV-2 virus, exerted profound and far-reaching impacts on global healthcare and the economy. It is now widely acknowledged that the endothelium plays a pivotal role in its pathogenesis and manifestations (115, 116). Intracellular penetration of SARS-CoV-2 into human cells occurs via union of its spike protein to the angiotensin converting enzyme 2 (ACE2) (117). While ACE2 receptors are ubiquitous, they are particularly overexpressed on the alveoli and the ECs (118), leading to an immediate interaction with the endothelium from the very initial stage of infection. As viral replication progresses, more severe symptoms may appear accompanied by a hyperinflammatory activation of host immunity. Key features at this stage are elevated acute phase reactants, such as C-reactive protein, D-dimer, and ferritin, as well as increased circulating cytokine levels. As discussed below, the endothelium is a primary contributor in sustaining and intensifying this maladaptive immune response (119, 120).

Critically ill COVID-19 patients develop pulmonary infiltrates leading to acute respiratory distress syndrome with an eventual need for respiratory support. The development of ARDS in COVID-19 involves a complex interplay of immune responses and inflammatory processes. Both direct (lung tissue-specific) and indirect (systemically triggered) mechanisms are involved in the pathogenesis of ARDS, but there is no evidence of a specific phenotype related to COVID-19 (121). The clinical diagnosis of ARDS is based on the Berlin definition (122) of sudden refractory hypoxemia and bilateral shadows in the lung fields. The endothelium plays a pivotal role in the spreading of inflammation and damage to the lung and the alveolar-capillary membrane, which culminates in fluid accumulation, compromised gas exchange, and pulmonary vasculature hypertension. The initial virus mediated pneumocyte injury triggers local cytokine production and, via paracrine cell communication, the alveolar capillary ECs sense the signaling distress (123). This activates the endothelium, which responds with the induction of a proinflammatory cell recruitment state, mediated by the expression of cell adhesion molecules (124). As a result, oxidative stress, endotheliitis and EC dysfunction ensue. Multiple mechanisms are involved in the switching of the endothelium into a hypercoagulative state, such as TF coagulation activation, platelet pro-aggregation due to increased release of VWF, NET-mediated thrombin generation and fibrinolytic shutdown. This is supported by the fact that COVID-19 patients present with increased circulating endothelial stress products (proinflammatory cytokine levels, VWF, soluble VCAM-1 and heparan sulfate) in correlation with disease severity (105). Also, biomarkers of complement activation, fibrinolysis inhibition, proangiogenic factors, and NET formation have shown to be significantly higher in the serum of COVID-19 patients (125–127). Several postmortem histological lung studies (128, 129) have widely evidenced the aforementioned mechanisms involved in the cascade of endothelial modifications, showing increased levels of inflammatory cytokines, such as IL-6 and TNF-α (130), and upregulation of ICAM-1 and VWF (131).

Other organs are often affected in the spectrum of COVID-19 manifestations, notably the cardiovascular system (132, 133). Thrombosis, coronary infarction and stroke are more frequently observed in the setting of critical illness (134). Further evidence pointing toward endotheliopathy as a predictor of COVID-19 morbidity and mortality emerges from the strong association between pre-existing cardiovascular risk factors (i.e., advanced age, hypertension, and obesity) and the likelihood of disease progression (135). In the brain, neurologic symptoms such as anosmia, ageusia or even encephalopathy were widely reported during the pandemics. In the skin, cutaneous lesions have also demonstrated endothelial activation. Histological studies evidenced complement-mediated vascular injury (136), particularly in severe COVID-19.

Current strategies primarily focus on high-efficacy antivirals and immunosuppressants. The combination of therapies targeting inflammation, coagulopathy, and endotheliopathy is a promising strategy to address disease complications. Many publications have explored other approaches to reduce ED, such as defibrotide (137), ACE inhibitors and ACE2 receptor blockers, statins, heparins, and direct oral anticoagulants. These therapies appear to be promising due to their pleiotropic mechanisms of action and their ability to regulate the endothelium (138).

Lastly, evidence of persistent endothelial dysfunction has been found in the subset of patients who develop long-term sequelae after acute infection. Elevated inflammatory markers, cytokine levels, and cytotoxic T cells subsist in convalescent patients (139), and vascular barrier injury is considered to be responsible for the disruption of normal organ physiology (140), leading to severely impairing systemic symptoms. While days of high infection incidence and overwhelming ICU admissions may be behind us, the implications of endothelial dysregulation remain relevant in the current era of persisting COVID-19. Therefore, endothelial protection remains a valid target for preventing both acute critical illness and long-term COVID-19 complications.

Our group of researchers has contributed to improving the knowledge of the endotheliopathy associated with COVID-19. We have provided evidence on biomarkers that may be useful for the stratification of disease severity and also to guide specific therapeutic strategies to prevent endotheliopathy progression. Some of these biomarkers help to differentiate COVID-19 endotheliopathy from the one that occurs in septic syndromes, in which ED is also a pathological substrate (120). Similarly, we have demonstrated that preeclampsia and severe COVID-19, which may be clinically similar, exhibit distinctive biomarker profiles related to ED, coagulopathy, and angiogenic imbalance. Therefore, differential diagnosis of these entities could be done based on these results.

## 8. The endothelium as a central player in thrombotic microangiopathies

Thrombotic microangiopathies (TMAs) are a group of disorders characterized by microangiopathic hemolytic anemia and ischemic organ dysfunction, resulting in a wide spectrum of symptoms. The most commonly affected organs in TMAs are the brain, kidneys, and gastrointestinal system (141). Although uncommon, these are life-threatening conditions that require urgent management (142). ED is the common underlying mechanism among different forms of TMAs, leading to the microvasculature thrombosis observed histologically (143).

Classically, primary TMAs have been classified according to the identification of the following pathogenic mechanisms: thrombotic thrombocytopenic purpura (TTP), mediated by a deficiency in the activity of ADAMTS-13 enzyme; typical hemolytic uremic syndrome, caused by a Shiga-toxin–producing Escherichia coli (STEC-HUS); and primary atypical HUS (aHUS), due to the dysregulation of the alternative complement pathway (ACP) (142).

Thrombotic thrombocytopenic purpura is characterized by ADAMTS-13 deficiency, resulting in a deficient excision of the ultra-large VWF multimers presented in the VWF molecule on ECs, causing platelet adhesion and aggregation with rapid generation of disseminated microthrombi. However, evidence generated from clinical, *in vitro*, and *in vivo* studies suggests that ADAMTS-13 deficiency may be a necessary but not sufficient condition to induce TTP. Weibel–Palade bodies (WPBs) are endothelial-specific organelles that contain molecules involved in the regulatory functions of the endothelium, such as ultra-large VWF multimers (proaggregating), P-selectin (proinflammatory) or Endothelin-1 (vasoconstricting). The “second hit” model suggests that in TTP, besides ADAMTS-13 deficiency, endogenous (antibodies or cytokines) and/or exogenous (virus or drugs) factors induce endothelial activation leading to an uncontrolled WPBs degranulation and, finally, to endothelial dysfunction (144).

In STEC-HUS, Shiga-toxin (Stx) is thought to be the key element in the pathogenesis of ED through several mechanisms. Stx induces the production of adhesive molecules (E-selectin, ICAM-1, and VCAM-1) and chemokines (MCP-1, IL-8, and fractalkine), leading to the adhesion of leukocytes to cultured human ECs. Moreover, Stx induces rapid release of ultra-large VWF multimers inhibiting its cleavage by ADAMTS-13, therefore enhancing platelet adhesion and clot formation in the microvasculature. In addition, Stx modifies gene expression, with mRNA production, and release of chemokines and cytokines that may aggravate ED (145, 146).

As mentioned before, dysregulation of the ACP occurs as the primary event in aHUS, prompting the activation of the terminal complement phase and the deposit of the lytic complex C5b-9 on the EC surface (146). Eculizumab, a humanized monoclonal antibody against C5, and ravulizumab, a long-acting C5 inhibitor, are first-line treatments for aHUS (147). In this regard, it has been observed that the measurement of C5b-9 deposits on ECs constitutes a reliable tool to explore the complement system dysregulation in aHUS, as well as to monitor the response efficiency to eculizumab treatment in these patients (83).

Although aHUS is the prototype of complement-mediated TMAs, the contribution of dysregulated complement activation to ED has been widely demonstrated in other TMA forms. Increased levels of soluble C5b-9 (sC5b-9) have been detected in patients with acute TTP, probably due to the activation of the classical complement pathway by immunocomplexes of ADAMTS-13 and anti-ADAMTS-13 antibodies (146). In STEC-HUS, the ED is caused by different mechanisms driven by Stx, including ACP activation, resulting in increased levels of C3a, Bb, and sC5b-9 during the active phase of the disease. In this regard, eculizumab has been employed for the treatment of children and adults with STEC-HUS, with no systematic assessment of its efficacy or safety (145).

Secondary forms of TMAs may occur in multiple clinical settings: autoimmune diseases, cancer, pregnancy, solid organ and HCT, certain medications, and infections (141). The underlying pathogenic mechanism has been attributed to a direct ED conferring a more procoagulant and proinflammatory phenotype in ECs, inducing a “second-hit” in which complement activation and its perpetuation occurs, aggravating TMA (148). Among them, TMAs after kidney transplantation should be highlighted due to the well-known involvement of ED in its pathogenesis, which can occur due to the combination of multiple triggers: ischemia-reperfusion injury, immunosuppressive drugs (calcineurin and m-TOR inhibitors), viral infections (mainly cytomegalovirus), and acute or chronic humoral rejection (148).

Thrombotic microangiopathies differential diagnosis is a major challenge due to their variable clinical presentation and the absence of pathognomonic histological findings or specific biomarkers (141). Moreover, differentiation between primary or secondary forms may be difficult in clinical practice because triggering factors, such as infections or drugs, are often identified in patients with primary aHUS (142). Therefore, there is an urgent need for new diagnostic tools, based on functional and genetic studies, to assess the involvement of complement dysregulation in the pathogenesis of the different forms of TMAs (146). In this regard, the use of complement-targeted therapies in patients with secondary refractory TMAs to traditional therapy could be useful. The duration of the treatment should be tailored based on the presence of complement abnormalities and response to therapy (149).

## 9. Hematopoietic stem cell transplantation and endothelial damage

Hematopoietic stem cell transplantation is the best-known form of cellular therapy, widely used for the treatment of malignant and non-malignant hematologic, metabolic, or autoimmune disorders (150). During the last decades, HCT has experienced significant improvements in terms of donor selection, in allogeneic HCT (allo-HCT), and treatment refinement in both, allo- and autologous HCT (auto-HCT) (151, 152), allowing the expansion of HCT to older adults or patients with comorbidities. More recently, the scope of cellular therapy has expanded through the emergence of immunotherapies based on the cytotoxic effect of autologous cells, as CAR-T cells and tumor-infiltrating lymphocytes (TIL), for refractory/relapsed hematological malignancies and solid tumors, respectively. Despite the curative potential of cell based therapies, there are associated complications that may compromise the success of the treatment. In these complications, the endothelium seems to play a main role.

Sinusoidal obstruction syndrome (SOS), formerly known as veno-occlusive disease, was the first post-HCT complication where endotheliopathy was proven as its pathophysiological substrate and targeted for its treatment (153, 154). Consecutively, growing evidence points to endothelial dysfunction underlying other highly incident HCT-related complications, such as acute graft-versus-host disease (aGVHD) (155, 156), and the main CAR-T cell-related toxicities (157, 158).

Endotheliopathy has not only shown to be involved in the pathogenesis of the complications of cellular therapies but also to be the result of different harms toward ECs before and during the treatment. Mainly due to drugs used during the induction or consolidation chemotherapeutic schemes (159, 160) or the ones used for the conditioning treatment before and after the infusion of the autologous or allogeneic cells (161–165). Consequently, it is essential to understand the involvement of the endothelium and other associated pathways in the pathogenesis of cellular therapies-complications in order to better-stratify risk patients and develop targeted treatments and preventive strategies.

The HCT treatment itself induces endothelial dysregulation, leading to a hypercoagulable state. Studies demonstrate that while procoagulant molecules increase, levels of the main natural-anticoagulant molecules decrease in the context of HCT (166, 167). Furthermore, the innate and adaptative immune reactions, the PAMPs resulting from infections occurring during the HCT process, together with the toxic agents included in the preparative regimens, have been identified as *noxa* toward the endothelium.

Endothelial dysfunction after HCT has multiple origins and varies according to the time after HCT and anatomical location. In most cases, it implies increased leukocyte adhesion and transmigration, molecule extravasations, platelet activation, and cytokine liberation (168). The ED occurring after HCT and derived from the mentioned stressors would consist of the following: (1) increased synthesis of Ang-2, which is involved in endothelial inflammation increasing its permeability (169), (2) overexpression of adhesion molecules (such as ICAM-1, VCAM-1, E-selectin, and P-selectin), responsible for leukocyte recruitment and transmigration through the endothelium (170), (3) dysregulation of the vascular tone, since the endothelial synthesis of NO and prostacyclin is reduced, and (4) elevation of angiogenic molecules, such as vascular endothelial growth factor A (VEGFA), fibroblast growth factor 2 (FGF2), and Ang-2, molecules that have their respective receptors (VEGFR1 and VEGFR2, FGR1, and TIE-2) (171).

The complex link existing between endothelial activation and progression to endothelial dysfunction occurring during the HCT process has been investigated in different *in vitro* and pre-clinical studies. Results obtained indicate that ECs are activated and damaged by different factors, including drugs used in the conditioning regimen, radiotherapy, cytokines released by the injured tissues, endogenous microbial products that translocated through damaged mucosal barriers, immunosuppressants in allo-HCT, the engraftment process itself, and allo-reactivity (163, 168, 172, 173). Studies by our group and others, using an *in vitro* model of ED, allowed the investigation of the specific responses of the endothelium to controlled and well-known stimuli at different stages of the HCT, and also to molecules associated with HCT, such as lipopolysaccharide or TNF-α (174).

In the HCT setting, the ED occurring since the start of the conditioning regimen and during the post-transplant process is involved in a group of early and potentially life-threatening post-HCT endothelial complications (175, 176). These events generally appear during the first 100 days after the stem cell infusion, their diagnosis is mainly based on medical signs and symptoms, and all of them seem to begin at the capillary level and result from an endothelial dysfunction occasioned by the administration of chemotherapy, calcineurin inhibitors, G-CSF, infections, and allogeneic-derived reactivity (12, 168, 177).

Historically, SOS was the first complication in which the role of ED was proposed. Nevertheless, there is increasing evidence of the implication of the endothelium in the pathophysiology of other post-HCT vascular endothelial complications, such as engraftment syndrome, capillary leak syndrome, transplant-associated thrombotic microangiopathy (TA-TMA), GVHD, and the vascular idiopathic pneumonia syndrome.

Several groups, in which ours is included, have investigated the presence of circulating biomarkers for diagnosis and prognostication of post-HCT vascular endothelial complications (177–180). In general, the majority of the studies show increased levels of ED biomarkers, especially in patients with HCT complications (179, 181–183). The complexity of the diagnosis and clinical management required to treat post-HCT vascular endothelial complications enhances the need to increase the knowledge of predictors and clinical manifestations for the early detection of these syndromes to decrease mortality after HCT (168). Furthermore, the increasing knowledge of the physiopathology of these complications opens up potential pharmacologic interventions to prevent and treat ED and, therefore, to improve the outcome of patients receiving HCT.

## 10. Endotheliopathy in CAR-T cell immunotherapy

Treatment with CAR-T cells has risen as a viable and safe procedure for the treatment of relapsed/refractory hematological malignancies. CAR-T cell technology is based on the cytotoxic effect of T lymphocytes of the patient modified *in vitro* to target antigens present in tumoral cells. This immunotherapy has proven to be effective for the treatment of acute lymphoblastic leukemia and lymphomas expressing CD19 antigen and for myeloma patients when targeting B-cell maturation antigen. To date, only a few therapeutic schemes and constructs have been approved by Food and Drug Administration and European Medicines Agency (184, 185) whereas many others are still being under assessment in clinical assays (186). Despite the promising remission rates of these novel therapies, cytokine release syndrome (CRS) and immune effector cell-associated neurotoxicity syndrome (ICANS), are highly incident toxicities, and potentially life-threatening (187–189). Growing evidence sustains that endotheliopathy underlies and promotes the onset of these toxicities. However, not all endothelial dysfunction can be attributed to the administration of the construct but to different *noxa* before and during immunotherapy.

The use of conditioning, or lymphodepletion, before CAR-T cell infusion has proven to enhance the *in vivo* expansion of the construct, and both its engraftment and anti-tumoral function (190–192). The most commonly used drugs are cyclophosphamide (Cy) and fludarabine (Flu) in combination. Both, Cy and Flu, have shown to cause deleterious effects on ECs (173, 193) in *in vitro* assays. In fact, Flu has been observed to increase the incidence of CAR-T related toxicities (194, 195). Nevertheless, the individual impact of these drugs, *in vivo*, and the incidence of endothelial-related complications has to be elucidated.

Up to 80% of patients treated with CAR-T cell immunotherapy present CRS of any grade, clinically ranging from fever ± hypoxemia, hypotension, capillary leak and/or signs of specific-organ toxicity, depending on the severity of the case (196). Less incident, ICANS can be suspected by a wide range of symptoms and signs, such as headache, cognitive or motor impairment, delirium and seizures. Since ICANS is predisposed in the vast majority of cases by severe/early onset CRS (157, 197), the pathways involved in their development seem to be common. For this reason, the pathophysiology of CRS and ICANS will be reviewed altogether except when otherwise specified.

Clinically, different risk factors have been associated with an increased risk of developing CRS and ICANS: lymphodepletion schemes containing Flu, high burden/bone marrow involvement of the basal disease, and infusion of high doses of the CAR-T cell construct leading to high peaks of *in vivo* proliferation (188, 194). Biologically, elevations of proinflammatory cytokines (IL-6, interferon-γ, and TNF-α) after the construct infusion were firstly reported as the potential cause of CRS/ICANS in correlation with the clinical severity (187, 198).

Endothelial dysfunction appearing as a consequence of the mentioned cytokine storm, among other causes (199), has been hypothesized as a relevant pathway in the development of CAR-T cell-related toxicities (200). In the specific context of ICANS, the increased permeability within the endothelium of the blood-brain barrier was proven after observing the presence of CAR-T cells on the cerebrospinal fluid (157, 201–203). Recently, scores based on indirect biomarkers of endotheliopathy, such as EASIX or modified EASIX, have demonstrated to be reliable tools to predict the incidence or severe CRS and/or ICANS and their related decrease of the progression-free survival (204–207). Similar to other diseases with ED (112, 208, 209), innate immune activation and hemostasis imbalance are linked pathways also altered in CAR-T cell patients developing toxicities (210–213).

Coagulopathy is an underestimated adverse effect of CAR T-cell therapies (214) that usually derives from severe CRS or ICANS (157, 211). While isolated changes in coagulation parameters, such as elevation in D-dimer, increase in fibrinogen degradation product, decrease of fibrinogen levels and prolongation of activated partial thromboplastin time (aPTT), can be observed in a high proportion of patients (215, 216), the analytical and clinical phenotype of DIC occurs only in cases of high-grade toxicities and is related to an increase of the non-relapse mortality (215). More specifically, a recent study has described increased prothrombin time (PT) and aPTT, fibrinogen, D-dimer, factor VIII (FVIII) and VWF antigen levels in ≥grade 2 CRS. The manifestation of ICANS was associated with elevated PT, D-dimer, FVIII and VWF antigen levels and decreased fibrinogen and platelet count (207). Moreover, patients with high-grade ICANS were found to present higher levels of Ang-2 and VWF, lower levels of ADAMTS-13 metalloprotease and loss of VWF high molecular weight multimers than patients with lower severity grades (157, 198, 200). Although the consumptive mechanism seems to be the predominant one for the development of DIC, impairment of the liver function has also been described as an early indicator (217).

Recently, we have demonstrated that different circulating biomarkers of endotheliopathy, innate-immunity activation, hemostasis alterations and fibrinolytic imbalance may be good early-predictors of severe CRS/ICANS (ST2, Ang-2, and NETs). Also, the use of some of these biomarkers could be a feasible discriminating tool for the differential diagnosis between CAR T-cell-related severe toxicities and sepsis (Ang-2, NETs, sC5b-9, VWF antigen, and PAI-1 antigen) (213).

## 11. Pharmacological armamentarium

Targeted treatments against the noxa responsible for endothelial activation and dysfunction is the ideal first line therapy, though, in most cases we do not have that kind of treatments available. In terms of pharmacological armamentarium targeting the endothelium, different options are under assessment. For instance, statins, vitamin C, direct oral anticoagulants, heparins, and N-acetylcysteine are cheap and safe drugs that have demonstrated a protective effect toward the endothelium by reducing the oxidative stress (218–224). The use of drugs able to restore the anti-inflammatory and anticoagulant properties of the endothelium, such as defibrotide, activated protein C, recombinant TM, and Ang-1 levels (an endothelium stabilizer molecule as opposed to Ang-2) have shown promising effects in *in vitro* and in murine models of endothelial dysfunction (225–231). However, disappointing results were found in clinical trials using activated protein C for severe sepsis, highlighting the complexity of the mechanisms involved in ED. In addition, the use of complement inhibitors, such as eculizumab and ravulizumab, in diseases in which terminal complement activation prevails like aHUS, has shown to play a prominent role (83, 147). Other compounds, like TNF-α, IL-1β, and ACE2 receptor blockers, ACE inhibitors or SSRIs, used in synergy with other treatments (i.e., those for CAR T-related toxicities, COVID-19 or major depression) could be worth exploring in clinical trials as potential useful novel therapies in the specific context in which ED is implied (232).

## 12. Conclusion and future perspectives

The endothelium is an endocrine organ that plays essential functions in maintaining homeostasis. It regulates the vascular tone, hemostasis and fibrinolysis; it shows anti-inflammatory and anticoagulant actions; and it participates in angiogenesis, among other functions. The failure of the endothelial adaptability to the different circulating stimuli, independently of their nature, may cause in the loss of endothelial integrity and function, which is critical for the development of cardiovascular disease.

Endothelial damage, and alterations of several linked pathways, are increasingly being proposed as a pathophysiological substrate for different pathologies and cell therapy complications. The knowledge generated during the last years has promoted the development of panels composed of ED biomarkers for the early prediction of these complications, to stratify their risk, and to facilitate their follow-up. In this regard, new and old therapeutic and prophylactic strategies focused on endothelial protection are being proposed. However, their impact on the incidence of complications and non-relapse mortality should be further explored.

More basic research is needed to elucidate the whole bunch of mechanisms by which the endothelium becomes dysfunctional in a variety of pathological conditions, and more investment in clinical assays is necessary to demonstrate the effect of potentially useful drugs to prevent and treat the endothelium. Additionally, considering that the endothelium is the biggest organ in the body, probably followed by the gastrointestinal tract, we are convinced that the future investigations on the endothelium should consider the crosstalk between both organs, in which the microbiome could be cornerstone (233).

## Author contributions

MP: Conceptualization, Formal analysis, Investigation, Supervision, Writing – original draft, Writing – review and editing. AM-C: Writing – original draft, Writing – review and editing. MS: Writing – original draft, Writing – review and editing. SE-S: Conceptualization, Writing – original draft, Writing – review and editing. JM-S: Writing – original draft, Writing – review and editing. BD: Writing – original draft, Writing – review and editing. MR: Writing – original draft, Writing – review and editing. EG-O: Writing – original draft, Writing – review and editing. SF: Writing – original draft, Writing – review and editing. HV-C: Writing – original draft, Writing – review and editing. LY: Writing – original draft, Writing – review and editing. FC: Writing – original draft, Writing – review and editing. MN: Writing – original draft, Writing – review and editing. MD-R: Conceptualization, Data curation, Formal analysis, Funding acquisition, Investigation, Methodology, Project administration, Resources, Software, Supervision, Validation, Visualization, Writing – original draft, Writing – review and editing.
